# Supplementary material for: Targeted suppression of siRNA biogenesis in Arabidopsis pollen promotes triploid seed viability
Source: Nat Commun. 2024 May 30;15:4612. doi: 10.1038/s41467-024-48950-6 (PMC11139921; doi:10.1038/s41467-024-48950-6)
Supplement: Supplementary file 1 — Supplementary Information [file 41467_2024_48950_MOESM1_ESM.pdf]

Targeted suppression of siRNA biogenesis in Arabidopsis pollen promotes triploid seed viability

Kannan Pachamuthu[1,2], Matthieu Simon[1] and Filipe Borges[1,*]

[1] Université Paris-Saclay, INRAE, AgroParisTech, Institut Jean-Pierre Bourgin (IJPB), 78000, Versailles, France.

[2] Present address: School of Biosciences and Technology, Vellore Institute of Technology, Vellore, 632014, Tamil Nadu, India.

[*] Corresponding author: filipe.borges@inrae.fr

SUPPLEMENTARY INFORMATION

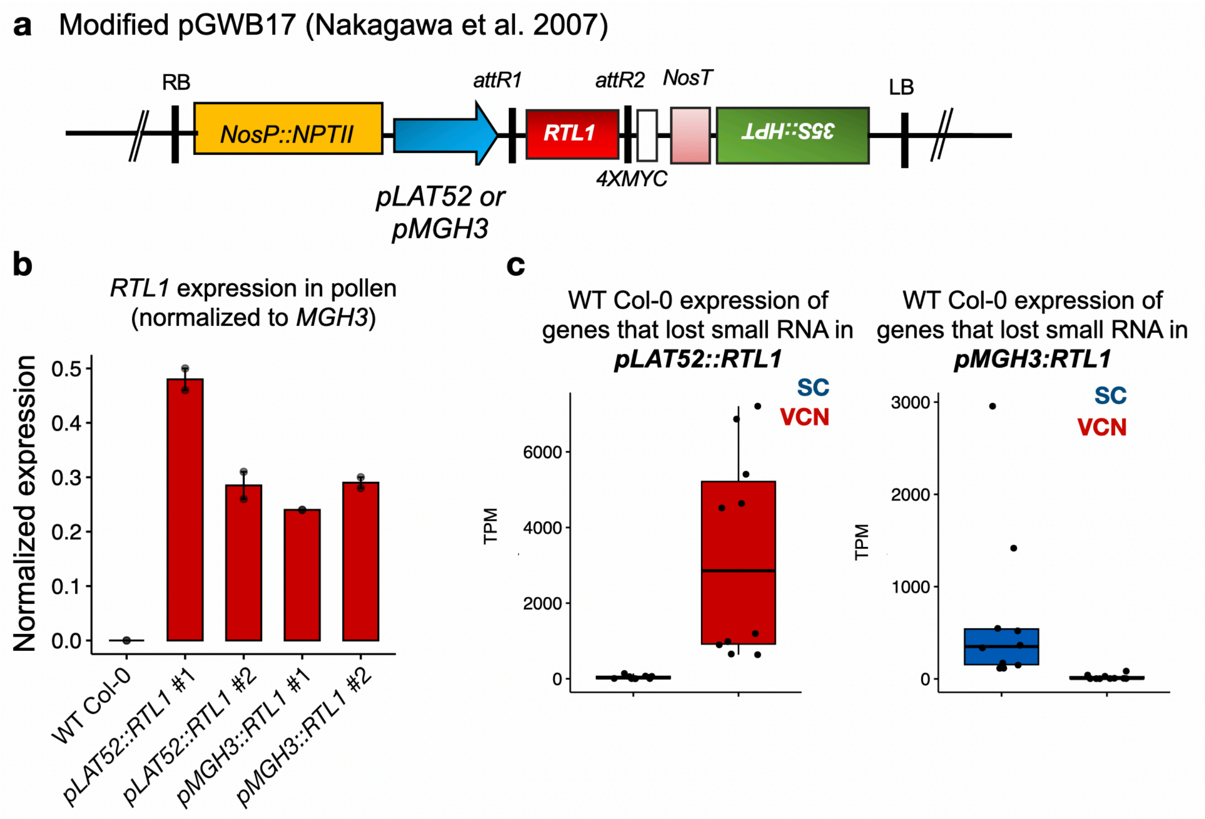

Supplementary Fig. 1 - Ectopic expression of *RTL1* in pollen. a, A modified version of the pGWB17 plasmid [1] containing the genomic sequence of *RTL1* (*AT4G15417*) was generated by replacing the 35S promoter by the *LAT52* or *MGH3* promoters. **b**, Stable *RTL1* expression in pollen was confirmed by RT-qPCR in two independent transgenic lines that were selected as homozygous for each transgene in the T_3 generation. Dots and bars represent expression level of individual replicates and mean values (n=2), respectively, error bars represent the standard error, and *RTL1* expression was normalized to the levels of the endogenous *MGH3* gene that is specifically expressed in pollen. **c**, Protein-coding genes that lost siRNAs in *pLAT52::RTL1* pollen are mainly expressed the vegetative cell nucleus (VCN), while genes that lost siRNAs in *pMGH3::RTL1* pollen are expressed in sperm cells (SC), confirming the specificity of our experiment. TPM is transcripts per million, and expression data of pollen nuclei is from Borg et al. (2021)[2]. Boxes represent the interquartile range (IQR) showing the lower (Q1) and upper (Q3) quartiles surrounding the median (central line), and whiskers represent the minimum (Q1 –1.5*IQR) and maximum (Q3 + 1.5*IQR) values. Source data are provided as a Source Data file.

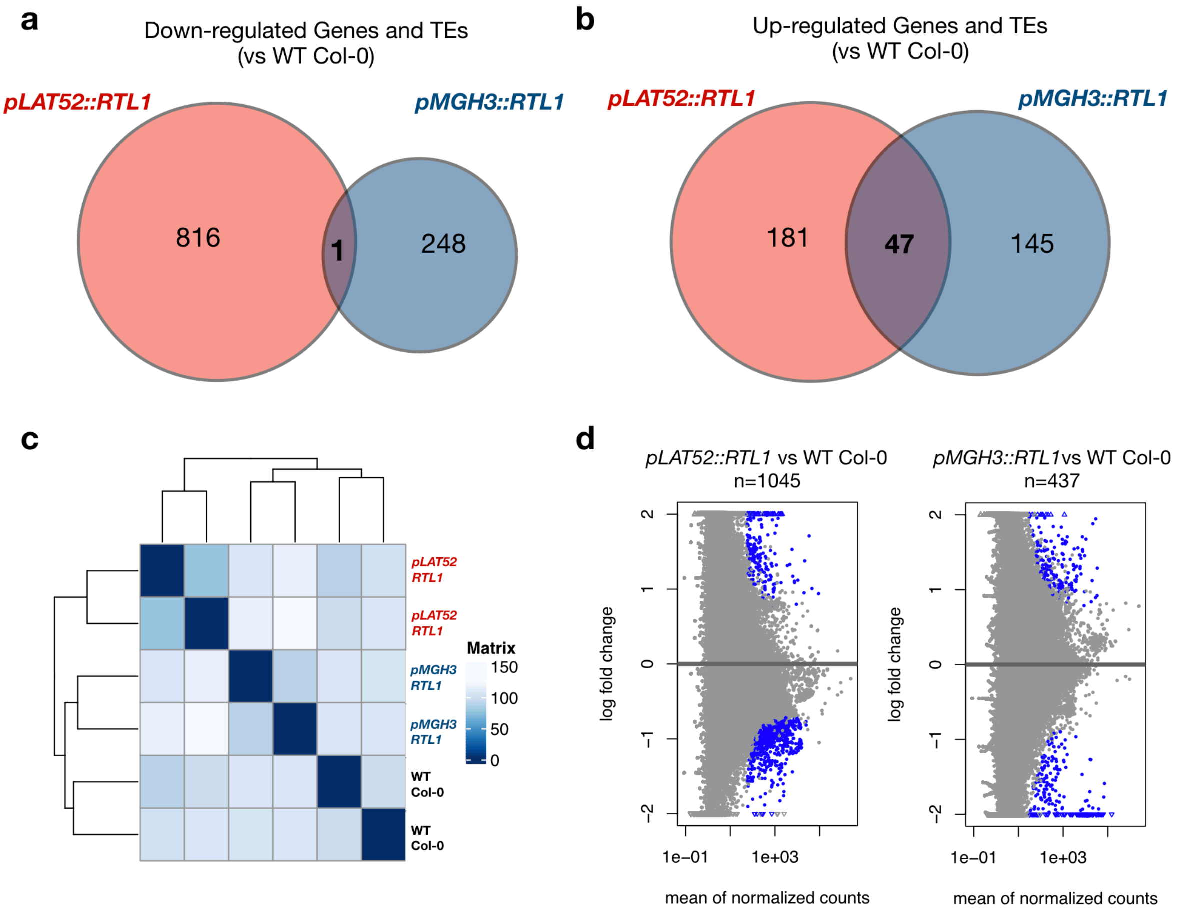

Supplementary Fig. 2 - Small RNA sequencing of *pLAT52::RTL1* and *pMGH3::RTL1* pollen. a, Venn diagram shows small overlap between genes and transposable elements (TEs) with significantly reduced siRNA levels in *pLAT52::RTL1* and *pMGH3::RTL1* pollen, as compared to wild-type control (WT Col-0). **b**, Venn diagram shows partial overlap between genes and TEs with up-regulated siRNA levels in *pLAT52::RTL1* and *pMGH3::RTL1* pollen (vs. WT Col-0). The statistical significance of the observed overlap was calculated using the R package SuperExactTest.[3] **c**, Small RNA datasets were transformed using variance-stabilizing transformation (VST) for sample clustering and visualization of sample-to-sample distances, which are represented by the scale gradient. The heatmap of this distance matrix shows an overview of similarities and dissimilarities between all datasets. **d**, MA plots highlight the number of genes and TEs differentially expressed in *pLAT52::RTL1* and *pMGH3::RTL1* pollen as calculated by DESeq2 (P <0.05), which are marked as blue dots. Source data are provided in Supplementary Data 1.

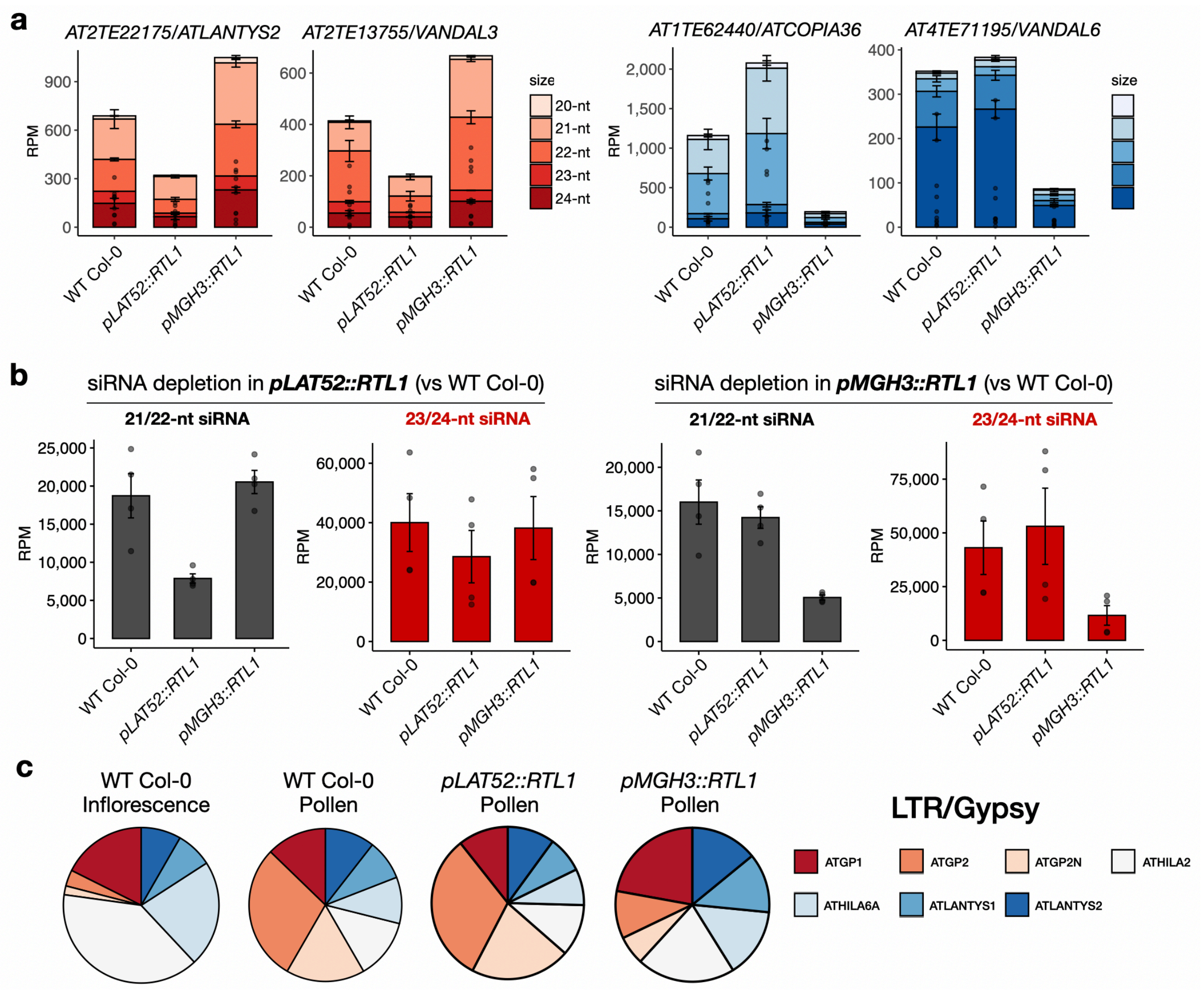

Supplementary Fig. 3 - Ectopic expression of *RTL1* in pollen suppresses siRNA biogenesis from TEs. a, Examples of individual TEs producing siRNA in WT Col-0 pollen, but showing significantly reduced levels in either *pLAT52::RTL1* or *pMGH3::RTL1* pollen. Error bars represent the standard error, and RPM is reads per million. **b**, Bar plots show the size distribution of siRNAs significantly depleted in *pLAT52::RTL1* and *pMGH3::RTL1* pollen. **c**, Pie charts represent the LTR/Gypsy families showing the highest siRNA levels in WT Col-0 pollen, to illustrate that siRNAs matching to *ATGP2* and *ATGP2N* are enriched in pollen as compared to inflorescence tissue, and depleted specifically in *pMGH3::RTL1* pollen. Dots and bars represent expression level of individual replicates and mean values (n=2), respectively, error bars represent the standard error, and RPM is reads per million. Source data are provided as a Source Data file.

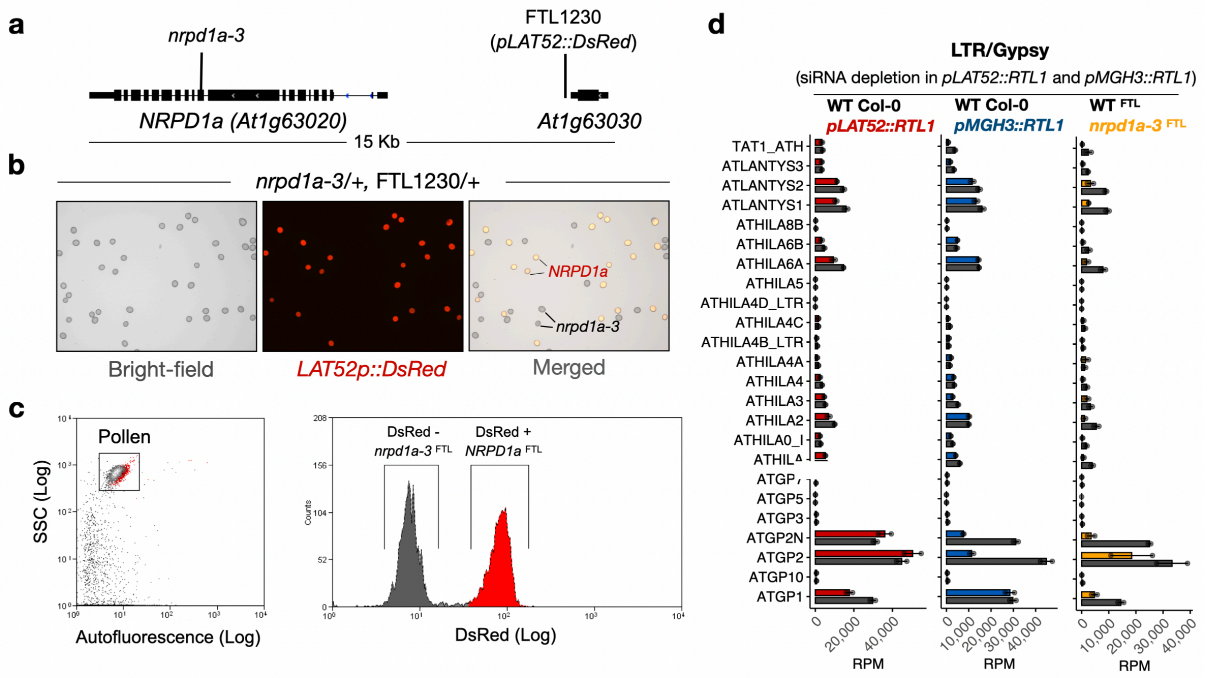

Supplementary Fig. 4 - FACS-based purification of *nrpd1a* pollen. a, A fluorescence-tagged line (FTL1230) was identified carrying the FTL transgene in close proximity to the *NRPD1a* (*AT1G63020*) gene that encodes the largest subunit of RNA Polymerase IV (Pol IV). The schematic depicts the localization of the FTL1230 transgene and the *nrpd1a-3* T-DNA mutant allele. **b**, A cross was performed between FTL1230 and *nrpd1a-3* mutant to obtain double heterozygous FTL1230/+, *nrpd1a-3*/+ F_1 plants showing approximately 50% of DsRed positive (DsRed +) pollen representing the WT allele of *NRPD1a* (WT^{FTL}), and approximately 50% of DsRed negative (DsRed -) pollen representing the mutant *nrpd1a-3* allele (*nrpd1a-3*^{FTL}). **c**, Fluorescence-activated cell sorting (FACS) was used to identify the two pollen populations that were first gated based on the elevated high angle scatter (SSC) and autofluorescence. WT (DsRed +) and mutant (DsRed -) pollen grains were subsequently purified based on their differences in DsRed signal. **d**, Small RNAs mapping to differentially expressed TEs within the LTR/Gypsy superfamily in *pLAT52::RTL1* and *pMGH3::RTL1* pollen are dependent on gametophytic activity of Pol IV. This is shown by the reduced levels in *nrpd1a-3*^{FTL} mutant pollen, showing that these siRNAs are produced specifically during pollen development. Dots and bars represent expression level of individual replicates and mean values (n=2), respectively, error bars represent the standard error, and RPM is reads per million. Source data are provided as a Source Data file.

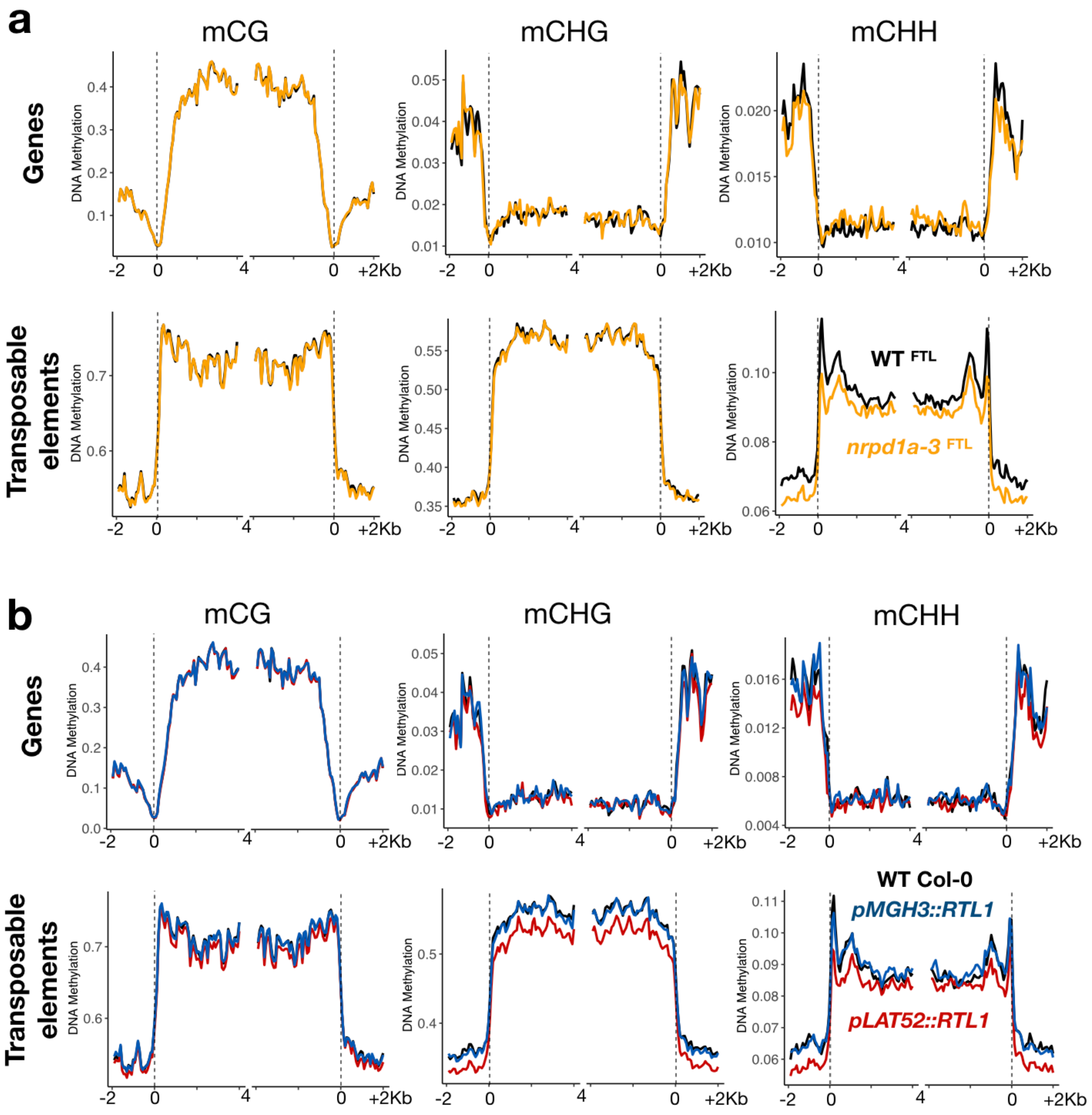

Supplementary Fig. 5 - Genome-wide DNA methylation levels in *pLAT52::RTL1* and *pMGH3::RTL1* pollen. a, Average CG, CHG, and CHH methylation levels in WT^{FTL} and *nrpd1a-3*^{FTL} pollen are plotted as 100-bp bins overlapping protein-coding genes or transposable elements annotated in the Arabidopsis TAIR10 reference genome, and aligned at the 5' and 3' ends (dashed lines). **b,** Average CG, CHG and CHH methylation levels at genes and TEs are also plotted for WT Col-0, *pLAT52::RTL1* and *pMGH3::RTL1* pollen, showing a reduction in non-CG methylation in *pLAT52::RTL1*. Source data are provided as a Source Data file.

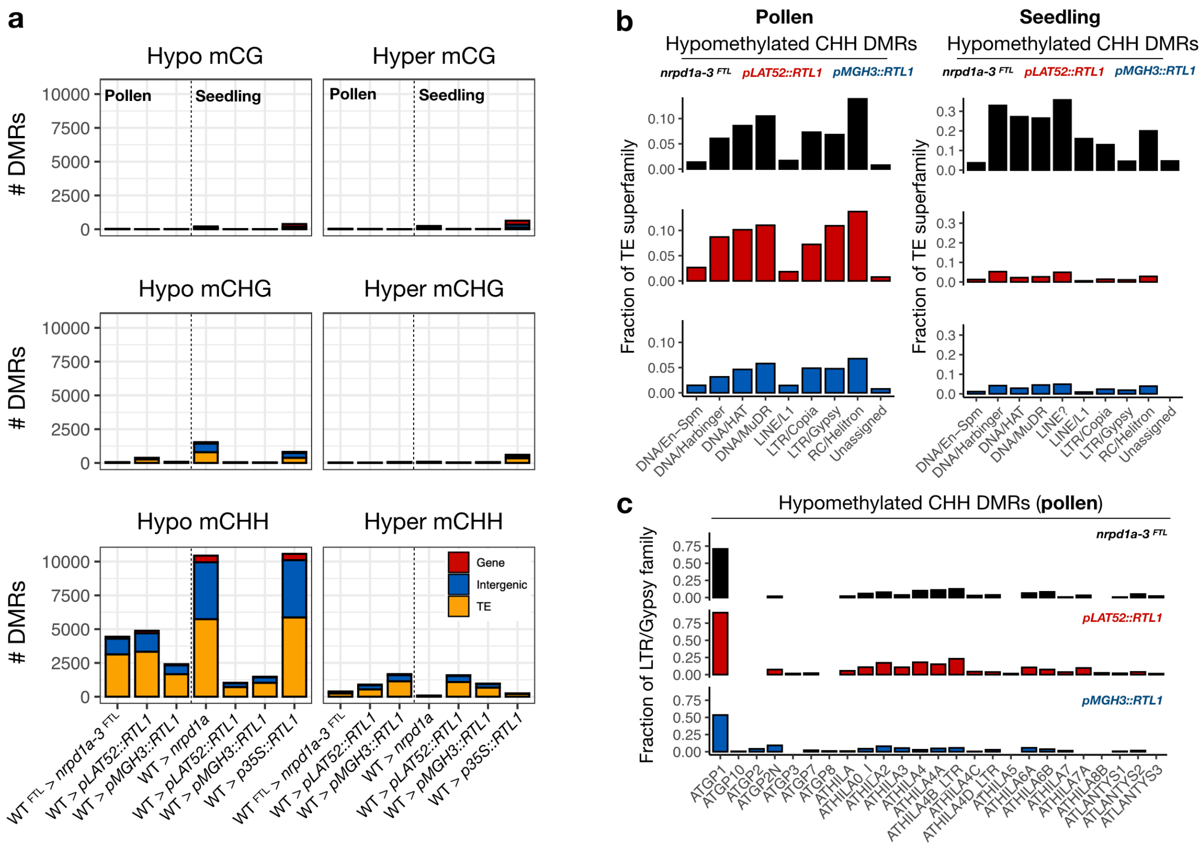

Supplementary Fig. 6 - Differentially methylated regions in *pLAT52::RTL1* and *pMGH3::RTL1* pollen and seedlings. a, Differentially methylated regions (DMRs) in *pLAT52::RTL1* and *pMGH3::RTL1* pollen (as compared to WT Col-0) are mainly hypomethylated in the CHH context and overlap with TEs. The number of DMRs detected for *pLAT52::RTL1* is similar to what is observed in *nrpd1a-3*^{FTL} pollen. **b**, Bar plots shows the fraction of TE superfamilies overlapping with hypomethylated CHH DMRs in *nrpd1a*, *pLAT52::RTL1* and *pMGH3::RTL1* pollen and seedlings. **c**, Bar plots show the fraction of LTR/Gypsy families overlapping with hypomethylated CHH DMRs in *nrpd1a*, *pLAT52::RTL1* and *pMGH3::RTL1* pollen. Source data are provided in Supplementary Data 2.

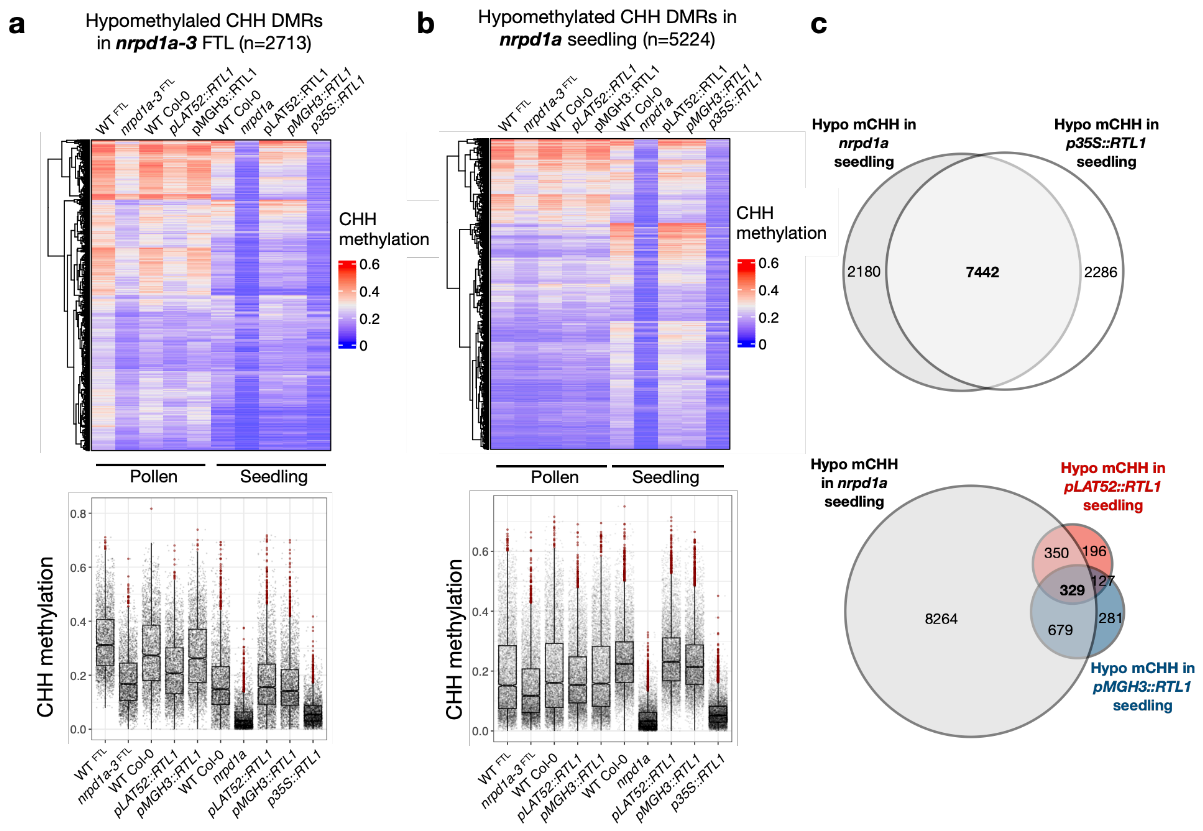

Supplementary Fig. 7 - CHH methylation levels at hypomethylated DMRs in *nrpd1a* pollen and seedlings. a, Heatmap and boxplot representation of differentially methylation regions (DMRs) in the CHH context between WT^{FTL} and *nrpd1a-3*^{FTL} pollen shows strong loss of CHH methylation in mutant pollen, which is comparable to what is observed when RTL1 is ectopically expressed in the vegetative cell (*pLAT52::RTL1*). **b,** Heatmap and boxplot representation of differentially methylation regions (DMRs) in the CHH context between WT Col-0 seedlings and *nrpd1a* homozygous mutant shows strong loss of CHH methylation in the mutant, which is comparable to what is observed when RTL1 is constitutively expressed by the 35S promoter (*p35S::RTL1*). Dots represent methylation level at individual DMRs, and boxes represent the interquartile range (IQR) showing the lower (Q1) and upper (Q3) quartiles surrounding the median (central line), and whiskers represent the minimum (Q1 –1.5*IQR) and maximum (Q3 + 1.5*IQR) values. Source data are provided as a Source Data file. **c**, Venn diagrams show the overlaps between hypomethylated regions in CHH context in *nrpd1a,* p35S::RTL1, *pLAT52::RTL1* and *pMGH3::RTL1* seedlings. The statistical significance of the observed overlaps was calculated using the R package SuperExactTest[3]. Source data are provided in Supplementary Data 2.

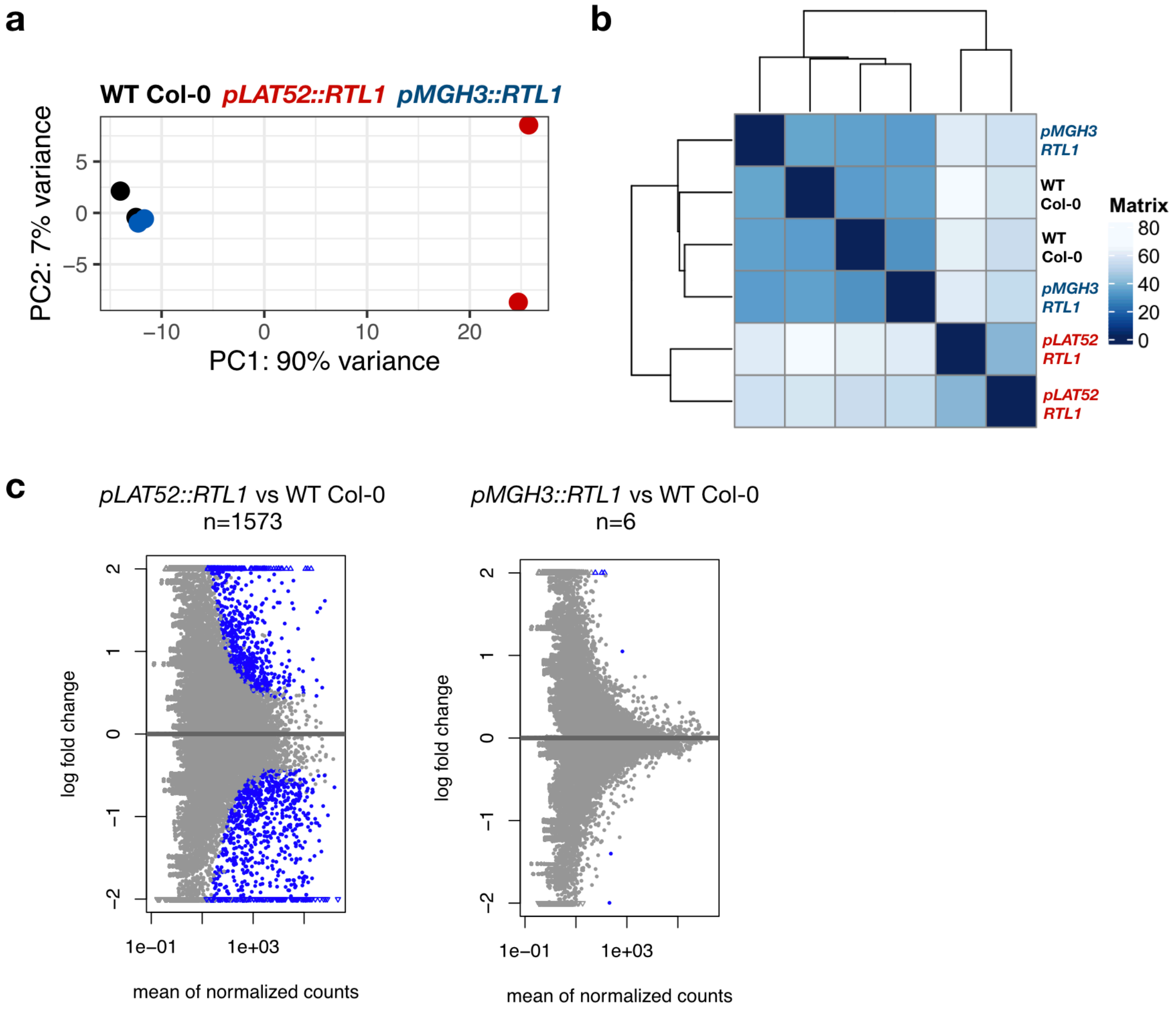

Supplementary Fig. 8 - RNA sequencing of *pLAT52::RTL1* and *pMGH3::RTL1* transgenic pollen. a, Principal component analysis after variance-stabilizing transformation shows the reproducibility of RNA sequencing experiments for two independent transgenic lines of each construct and wild-type Col-0 control. This also highlights differences between *pLAT52::RTL1* and *pMGH3::RTL1* datasets, as *pLAT52::RTL1* separates from WT Col-0 and *pMGH3::RTL1* along PC1. **b**, Datasets were transformed by variance-stabilizing transformation (VST) and used for sample clustering and visualization of sample-to-sample distances, which are represented by the scale gradient. The heatmap of this distance matrix shows an overview of similarities and dissimilarities between all datasets. **c**, MA plots highlight the number of genes and TEs differentially expressed in *pLAT52::RTL1* and *pMGH3::RTL1* pollen as calculated by DESeq2 (P <0.05), which are marked as blue dots.

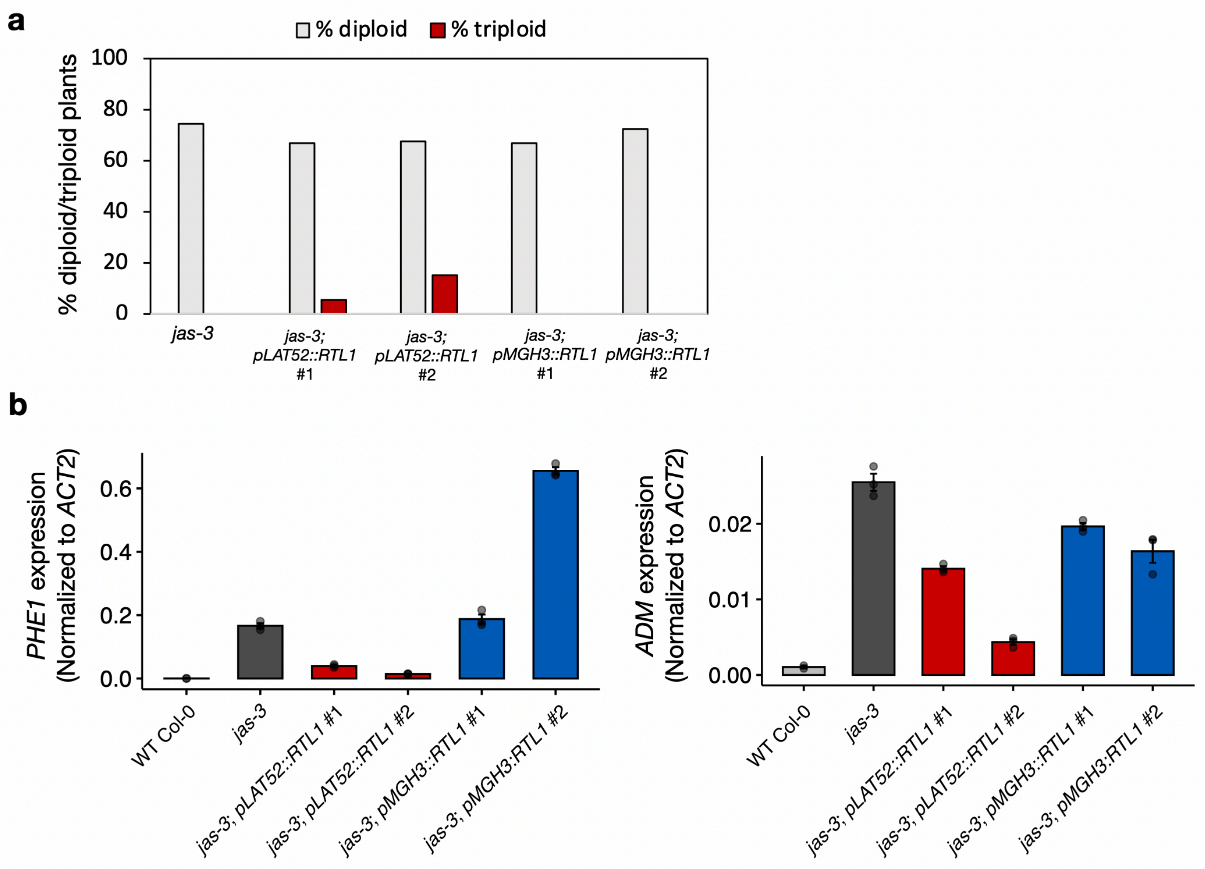

Supplementary Fig. 9 - Loss of siRNA biogenesis in the vegetative nucleus impacts triploid seed viability and genomic imprinting. a, The presence of viable triploid seeds in *jas-3*;*pLAT52::RTL1* lines was quantified by inspecting the presence of triploid plants in the selfed progeny, based on their fertility defects. **b**, Expression of the paternally expressed imprinted genes *PHE1* and *ADM* is up-regulated in *jas-3* mutant siliques 7 days after pollination, as compared to wild-type Col-0. This response is reduced in siliques of *jas-3*;*pLAT52::RTL1* lines, particularly for *PHE1* that was found down-regulated in two independent transgenic lines showing lower levels of seed collapse. Dots and bars represent expression level of individual replicates and mean values (n=2), respectively, error bars represent the standard error, and expression of *PHE1* and *ADM* was normalized to the levels of *ACT2*. Source data are provided as a Source Data file.

Supplementary Table 1 - Primers used in this study.

Name	Sequence (5' - 3')
Cloning	
pLAT52_HindIII_F	aatAAGCTTGACATACTCGACTCAGAAGGTA
pLAT52_XbaI_R	aatTCTAGATTTTTTTTGGTGTGTGTACTT
pMGH3_HindIII_F	aatAAGCTTTACTTCTCCGACCAAAAACTT
pMGH3_XbaI_R	aatTCTAGAGTGCGATTTCTTCGAGAGAAC
RT-qPCR	
RTL1_F	ATGGACTGCAACTCCACC
RTL1_R	GTTCTGTCATTGGATGGCTC
MGH3_F	TGTCCAAGGACATTCAGTTGGC
MGH3_R	CACGGAAGGGTTTATCAGAG
ACT2_F	GAGAGATTCAGATGCCCAGAAGTC
ACT2_R	TGGATTCCAGCAGCTTCCA
ADM_F	TTGAAAGAGTTTGCGGATGTG
ADM_R	AGGACCAACATTATGGTCATACC
PHE1_F	TTGGTGTAGCTCCTACTGTTGT
PHE1_R	AGCCTGGTATTGGAATTGAACC
Genotyping	
LBb1.3	ATTTTGCCGATTTCGGAAC
*nrpd1a-3*_F	GATCTGTTCAGCTTGCTCGTC
*nrpd1a-3*_R	TGCTTATGATGGCACTGTGAG
LB_T-DNA_FTL	GGCATGCAAGCTGATAATTC
FTL1230_F	GAATTCTCCAACACAGCTCAGG
FTL1230_R	AGATCCGCGTGTTTGAATTTCT
LB3	TAGCATCTGAATTTCATAACCAATCTCGATACAC
*jas-3*_SAIL_F	CACTTCAAATGGTGTTGCATG
*jas-3*_SAIL_R	TCTTCCCATTTTCACTCATGG

SUPPLEMENTARY REFERENCES

1. Nakagawa, T. *et al.* Development of series of gateway binary vectors, pGWBs, for realizing efficient construction of fusion genes for plant transformation. *Journal of Bioscience and Bioengineering* **104**, 34–41 (2007).
2. Borg, M. *et al.* Epigenetic reprogramming rewires transcription during the alternation of generations in Arabidopsis. *eLife* **10**, e61894 (2021).
3. Wang, M., Zhao, Y. & Zhang, B. Efficient Test and Visualization of Multi-Set Intersections. *Sci Rep* **5**, 16923 (2015).
