# Supplementary material for: Targeted suppression of siRNA biogenesis in Arabidopsis pollen promotes triploid seed viability
Source: Nat Commun. 2024 May 30;15:4612. doi: 10.1038/s41467-024-48950-6 (PMC11139921; doi:10.1038/s41467-024-48950-6)
Supplement: Supplementary file 3 — Description of Additional Supplementary Files [file 41467_2024_48950_MOESM3_ESM.pdf]

Description of Additional Supplementary Files

Supplementary Data 1 - Differentially expressed small RNAs from genes and TEs.

Supplementary Data 2 - Differentially Methylated Regions (DMRs).

Supplementary Data 3 - Differentially expressed genes and TEs.

Supplementary Data 4 - Summary of RNA sequencing and whole-genome bisulfite sequencing (WGBS).

Supplementary Data 5 - Statistical analysis.
