# Supplementary material for: Targeted suppression of siRNA biogenesis in Arabidopsis pollen promotes triploid seed viability
Source: Nat Commun. 2024 May 30;15:4612. doi: 10.1038/s41467-024-48950-6 (PMC11139921; doi:10.1038/s41467-024-48950-6)
Supplement: Supplementary file 9 — Reporting Summary [file 41467_2024_48950_MOESM9_ESM.pdf]

nature portfolio

Corresponding author(s): Filipe Borges

Last updated by author(s): Apr 24, 2024

Reporting Summary

Nature Portfolio wishes to improve the reproducibility of the work that we publish. This form provides structure for consistency and transparency in reporting. For further information on Nature Portfolio policies, see our Editorial Policies and the Editorial Policy Checklist.

Statistics

For all statistical analyses, confirm that the following items are present in the figure legend, table legend, main text, or Methods section.

n/a	Confirmed	
☐	☒	The exact sample size (*n*) for each experimental group/condition, given as a discrete number and unit of measurement
☐	☒	A statement on whether measurements were taken from distinct samples or whether the same sample was measured repeatedly
☐	☒	The statistical test(s) used AND whether they are one- or two-sided<br>*Only common tests should be described solely by name; describe more complex techniques in the Methods section.*
☒	☐	A description of all covariates tested
☒	☐	A description of any assumptions or corrections, such as tests of normality and adjustment for multiple comparisons
☐	☒	A full description of the statistical parameters including central tendency (e.g. means) or other basic estimates (e.g. regression coefficient) AND variation (e.g. standard deviation) or associated estimates of uncertainty (e.g. confidence intervals)
☐	☒	For null hypothesis testing, the test statistic (e.g. *F*, *t*, *r*) with confidence intervals, effect sizes, degrees of freedom and *P* value noted<br>*Give P values as exact values whenever suitable.*
☒	☐	For Bayesian analysis, information on the choice of priors and Markov chain Monte Carlo settings
☒	☐	For hierarchical and complex designs, identification of the appropriate level for tests and full reporting of outcomes
☒	☐	Estimates of effect sizes (e.g. Cohen's *d*, Pearson's *r*), indicating how they were calculated

Our web collection on statistics for biologists contains articles on many of the points above.

Software and code

Policy information about availability of computer code

Data collection	Summit 6.3 (Beckman Coulter)
Data analysis	bowtie-1.2.1.1, bedtools-2.26.0, samtools-1.10, fastx_toolkit-0.0.14, IGV_2.4.10, Bismark_v0.20.0, R-4.0.5, STAR-2.6.0c

For manuscripts utilizing custom algorithms or software that are central to the research but not yet described in published literature, software must be made available to editors and reviewers. We strongly encourage code deposition in a community repository (e.g. GitHub). See the Nature Portfolio guidelines for submitting code & software for further information.

Data

Policy information about availability of data

All manuscripts must include a data availability statement. This statement should provide the following information, where applicable:

- Accession codes, unique identifiers, or web links for publicly available datasets
- A description of any restrictions on data availability
- For clinical datasets or third party data, please ensure that the statement adheres to our policy

All sequencing datasets generated in this study are publicly available in the NCBI’s Gene Expression Omnibus under the accession number GSE231670. Expression profiling of Col-0 pollen nuclei is available in GSE155369. Source data are provided with this paper.

Research involving human participants, their data, or biological material

Policy information about studies with human participants or human data. See also policy information about sex, gender (identity/presentation), and sexual orientation and race, ethnicity and racism.

Reporting on sex and gender	N/A
Reporting on race, ethnicity, or other socially relevant groupings	N/A
Population characteristics	N/A
Recruitment	N/A
Ethics oversight	N/A

Note that full information on the approval of the study protocol must also be provided in the manuscript.

Field-specific reporting

Please select the one below that is the best fit for your research. If you are not sure, read the appropriate sections before making your selection.

- [x] Life sciences
- [ ] Behavioural & social sciences
- [ ] Ecological, evolutionary & environmental sciences

For a reference copy of the document with all sections, see nature.com/documents/nr-reporting-summary-flat.pdf

Life sciences study design

All studies must disclose on these points even when the disclosure is negative.

Sample size	Two independent transgenic lines were generated for each RTL1 construct. No statistical method was used to predetermine the sample size, and this sample size should be sufficient to validate the specificity of the approach.
Data exclusions	For sRNA-seq, only perfect-match reads were used for all downstream analysis. For differential expression analysis, features with low counts (<12) were excluded (DESeq2 default settings). For the identification of differentially methylated regions, only cytosines covered by a least 3 reads were used.
Replication	Two biological replicates were used for sRNA and mRNA sequencing, which has been proven sufficient to identify differentially expressed loci. Genome-wide bisulfite sequencing does not normally requires replicates do identify DMRs, which is still a widely accepted view in the field. Three biological replicates were used for triploid block analysis, which was necessary to provide robust statistical analysis of small differences in the levels of seed collapse.
Randomization	Not relevant because there are no treatment groups.
Blinding	Not relevant because there are no treatment groups.

Reporting for specific materials, systems and methods

We require information from authors about some types of materials, experimental systems and methods used in many studies. Here, indicate whether each material, system or method listed is relevant to your study. If you are not sure if a list item applies to your research, read the appropriate section before selecting a response.

Materials & experimental systems

n/a	Involved in the study	
x		Antibodies
x		Eukaryotic cell lines
x		Palaeontology and archaeology
x		Animals and other organisms
x		Clinical data
x		Dual use research of concern
	x	Plants

Methods

n/a	Involved in the study	
x		ChIP-seq
	x	Flow cytometry
x		MRI-based neuroimaging

Dual use research of concern

Policy information about dual use research of concern

Hazards

Could the accidental, deliberate or reckless misuse of agents or technologies generated in the work, or the application of information presented in the manuscript, pose a threat to:

No	Yes	
☒	☐	Public health
☒	☐	National security
☒	☐	Crops and/or livestock
☒	☐	Ecosystems
☒	☐	Any other significant area

Experiments of concern

Does the work involve any of these experiments of concern:

No	Yes	
☒	☐	Demonstrate how to render a vaccine ineffective
☒	☐	Confer resistance to therapeutically useful antibiotics or antiviral agents
☒	☐	Enhance the virulence of a pathogen or render a nonpathogen virulent
☒	☐	Increase transmissibility of a pathogen
☒	☐	Alter the host range of a pathogen
☒	☐	Enable evasion of diagnostic/detection modalities
☒	☐	Enable the weaponization of a biological agent or toxin
☒	☐	Any other potentially harmful combination of experiments and agents

Flow Cytometry

Plots

Confirm that:

☒ The axis labels state the marker and fluorochrome used (e.g. CD4-FITC).

☒ The axis scales are clearly visible. Include numbers along axes only for bottom left plot of group (a 'group' is an analysis of identical markers).

☒ All plots are contour plots with outliers or pseudocolor plots.

☐ A numerical value for number of cells or percentage (with statistics) is provided.

Methodology

Sample preparation	Pollen was purified by collecting open flowers into Eppendorf tubes, vortexing in 2 mL of 100-mM sodium phosphate buffer (pH 7) for 3 min, and filtering through a 50µm nylon mesh (CellTrics, Sysmex).
Instrument	MoFlo Astrios EQ sorter (Beckman-Coulter)
Software	Summit 6.3 (Beckman Coulter)
Cell population abundance	Approximately 500.000 pollen grains were collected. 50% of DsRed positive (DsRed +) pollen represents the WT allele of NRPD1a (WTFTL), and 50% of DsRed negative (DsRed -) pollen. Purity was confirmed based on transgene expression levels in RNA-seq data, and methylome analysis.
Gating strategy	Pollen populations are characterized by an elevated high angle scatter (SSC) and autofluorescence, which were then separated based on the differences in DsRed signal intensity.

☒ Tick this box to confirm that a figure exemplifying the gating strategy is provided in the Supplementary Information.
